# A Self-Administered Eating Behavior Scale for Patients With Heart Failure Living at Home: Protocol for a Mixed Methods Scale Development Study

**DOI:** 10.2196/60719

**Published:** 2024-10-18

**Authors:** Daisaku Kashiwakura, Akiko Hiyama, Masumi Muramatsu, Atsuko Hinotsu, Michiko Takeda, Norio Suzuki, Sachie Akiyama, Sayuri Kurihara, Keisuke Kida

**Affiliations:** 1 Graduate School of Nursing Sapporo City University Sapporo Japan; 2 Department of Nursing Faculty of Health and Medical Care Japan Healthcare University Sapporo Japan; 3 Department of Nursing Aishin Memorial Hospital Sapporo Japan; 4 Division of Cardiology, Department of Internal Medicine St. Marianna University School of Medicine Kawasaki Japan; 5 Department of Nursing Kawasaki Municipal Tama Hospital Kawasaki Japan; 6 Department of Nursing St. Marianna University School of Medicine Kawasaki Japan; 7 Department of Pharmacology St. Marianna University School of Medicine Kawasaki Japan

**Keywords:** heart failure, eating behavior, self-care, patient-reported outcome measures, International Classification of Functioning, Disability, and Health (ICF)

## Abstract

**Background:**

The prevalence of heart failure (HF) is increasing worldwide, with the associated mortality rates rising consistently. Preventing HF progression requires adherence to restricted sodium intake alongside sufficient and balanced nutritional consumption. For patients at home, preparing nutritionally balanced meals is essential, either self-assisted or with the aid of close individuals. Patients with HF frequently experience decreased exercise tolerance, depression, anxiety, and social isolation, which interfere with eating behaviors, leading to inadequate dietary habits. However, measures focusing on the determinants of eating behavior among patients with HF are currently lacking.

**Objective:**

This study aims to develop a self-administered scale to assess the eating behaviors of patients with HF living at home (Self-Administered Eating Behaviors Scale for Heart Failure [SEBS-HF]).

**Methods:**

This study encompasses 3 phases. Phase 1 involves identifying factors influencing eating behaviors in patients with HF. First, a literature review will be conducted using PubMed and CINAHL databases. The specified literature will be analyzed qualitatively and inductively. Additionally, verbatim transcripts obtained from semistructured interviews of patients with HF and medical experts will be qualitatively analyzed. Based on the Phase 1 results, a preliminary scale will be constructed. In Phase 2, cognitive interviews will be conducted with patients with HF and experts; the preliminary scale will be used to qualitatively evaluate its content validity. After validation, the scale will be used in Phase 3 to conduct a cross-sectional study involving patients with HF. In Phase 3, data will be collected from clinical records and self-administered questionnaires or scales. After conducting a preliminary survey, the main survey will be conducted. The reliability and validity of the scale will be assessed using statistical methods.

**Results:**

The first phase of this study commenced in September 2023, and by May 2, 2024, a total of 7 patients with HF and 6 expert professionals were enrolled as study participants. The draft creation of the scale will be completed in 2024, and the content validity evaluation of the draft scale is expected to be finished by early 2025. The third phase will begin its investigation in mid-2025 and is expected to be completed by late 2025, after which the SEBS-HF will be published.

**Conclusions:**

The development and use of this scale will enable a more comprehensive evaluation of the factors influencing eating behaviors in patients with HF. Thus, medical and welfare professionals should provide appropriate support tailored to the specific needs of patients with HF.

**International Registered Report Identifier (IRRID):**

DERR1-10.2196/60719

## Introduction

Heart failure (HF) is a clinical syndrome characterized by signs and symptoms resulting from a structural or functional impairment in ventricular filling or blood ejection [1]. HF is a global pandemic, with its prevalence steadily increasing globally, coupled with a consistent increase in associated mortality rates [2]. The prevalence of HF in Japan is estimated to be between 2.2% and 6.5% [3], with HF-related deaths accounting for 6.3% of all mortalities [4]. Given the context of a super-aged society, Japan is currently facing an HF pandemic [5].

Effective self-care for patients with HF primarily involves medication adherence, dietary habits, and physical activity [1]. Self-care measures, such as the European Heart Failure Self-care Behavior Scale (EHFScBS) [6] and the Self-Care of Heart Failure Index (SCHFI) [7], are widely recognized. A Japanese version of the EHFScBS has been developed and used [8]. Patients who adhered to appropriate self-care, as indicated by their EHFScBS scores, had significantly fewer hospitalizations due to all causes than those who did not [9]. The EHFScBS and SCHFI, which include dietary habits as part of self-care, primarily evaluate patient adherence.

Dietary recommendations for preventing HF progression and its associated adverse events are based on balancing sodium restriction with adequate nutrient intake [1]. Concerning low-sodium diets, large-scale randomized controlled trials have demonstrated that stringent sodium restrictions have limited benefits [10]. Therefore, a moderately low-sodium diet and adequate nutritional intake are recommended. However, a low-sodium diet often leads to inadequate intake of macronutrients and micronutrients [11,12]. HF comorbidities, including malnutrition, are known to affect prognosis adversely [13]. In addition, maintaining a balanced diet after discharge from the hospital is crucial to prevent HF progression. Home dietary management entails selecting suitable ingredients and meals, purchasing and preparing them, and making associated decisions. The term “eating behavior” broadly encapsulates these series of actions and decision-making processes [14].

The Brief Dietary Psychosocial Scale (BDPS) is an instrument that targets adolescents and adults, focusing on psychosocial factors and external environments that influence healthy dietary habits, such as the consumption of fruits, vegetables, fiber/whole grains, and fats [15]. The BDPS assesses whether individuals perceive the benefits they derive from a healthy diet, whether they are trying to maintain or change to a healthy diet, whether they are receiving social support for the diet, whether healthy food ingredients are physically and economically accessible, and whether they are enjoying following a healthy diet. The reliability of this scale has previously been evaluated in patients with HF [16].

The Dietary Sodium Restriction Questionnaire (DSRQ) is designed to assess attitudes, subjective norms, and perceived behavioral control related to dietary sodium restriction in patients with HF and hypertension [17]. Of the 3 DSRQ subscales, the subjective norms are predictive factors for adherence to a low-sodium diet [18].

The Burden Scale In Restricted Diets (BIRD) is a specific instrument developed to assess the burden of low-sodium diets in patients with HF [19]. This questionnaire comprises 14 candidate items for the following dietary-related domains: organization, pleasure, leisure, social life, vitality, and self-rated health.

The International Classification of Functioning, Disability, and Health (ICF) is a framework developed by the World Health Organization that categorizes and describes the health and disability status of individuals [20]. Under the ICF framework, eating behavior pertains to activities and participation domains. Patients with HF often experience decreased exercise tolerance [21], depression, and anxiety [22], which fall under the domains of body function and structure, and social isolation [23], included in the environmental factors domain. These conditions among patients with HF may hinder the eating behaviors necessary for proper home dietary management, potentially leading to acute decompensated HF.

Existing measures, such as the BDPS, DSRQ, and BIRD, focus on factors influencing healthy eating and low-sodium diets but lack a perspective on eating behaviors and do not focus on the body functions and structures domain in the ICF, corresponding to decreased exercise tolerance in HF.

Despite these reasonable hypotheses, measures focusing on the eating behavior determinants among patients with HF are currently lacking. Therefore, this study aims to develop and validate the Self-Administered Eating Behaviors Scale for Heart Failure (SEBS-HF) to assess the dietary habits of patients with HF living at home.

## Methods

### Design and Study Population

This study was designed in accordance with the COSMIN (Consensus-Based Standards for the Selection of Health Measurement Instruments) Study Design checklist for patient-reported outcome measurement [24] and the COSMIN methodology for evaluating the content validity of patient-reported outcome measures [25]. The qualitative descriptive investigation in this study adheres to the guidelines outlined in the COREQ (Consolidated Criteria for Reporting on Qualitative Research) checklist [26].

The criteria for identifying patients with HF in this study are defined as follows. The eligible population includes patients aged ≥18 years with symptomatic HF (stage C), according to the American College of Cardiology/American Heart Association/Heart Failure Society of America guidelines [1]. Under the Japanese long-term care insurance system, individuals aged ≥65 years are assessed and classified into 7 levels of care needs based on their activities of daily living, cognitive function, and medical conditions. For patients aged ≥65 years, only those yet to receive long-term care certification or those who fall within the range of “Requiring Help 1” to “Long-term Care Level 1” will be included. This is because the SEBS-HF is aimed at home-based patients with HF who can self-manage their diet to some extent, focusing on individuals who are relatively independent but may still require minimal assistance.

The exclusion criteria are as follows: living in care facilities, assisted living facilities, or nursing homes; aged ≤65 years with a disability certificate unrelated to cardiac dysfunction; undergoing hemodialysis because of dietary restrictions different from those of patients with HF; chronic respiratory diseases because of symptoms similar to HF such as reduced exercise tolerance; and mental illnesses because of the potential impact on eating behaviors.

Patients with HF eligible for this study will be recruited from either outpatients or inpatients at the medical institutions where the coresearchers are affiliated and the medical institutions collaborating in this study. Specifically, patients who are attending or admitted to the cardiology departments of these medical institutions will be included.

Experts will be recruited through the network of the principal investigator and coresearchers, and snowball sampling of the initially recommended experts.

### Study Composition

#### Overview

This study is composed of 3 phases (Textbox 1).

**Composition of the study.**

**Phase 1: Scale draft creation using qualitative descriptive method**

- Extraction of factors influencing eating behavior through literature review
- Extraction of factors influencing eating behavior through semistructured interviews with patients
- Extraction of factors influencing patients’ eating behavior through semistructured interviews with experts
- Itemization and creation of the scale draft from the scale items obtained

**Phase 2: Evaluation of the scale draft’s content validity by patients and experts**

- Evaluation of the scale draft’s face validity and revision of the draft through semistructured interviews with patients
- Evaluation of the comprehensibility, relevance, and inclusiveness of the scale draft and revision of the draft through semistructured interviews with patients
- Evaluation of the relevance and inclusiveness of the scale draft and revision of the draft through semistructured interviews with experts

**Phase 3: Evaluation of the reliability and validity of the scale draft through a cross-sectional survey**

- Preliminary investigation
- Primary investigation
- Analysis of the data obtained from the primary survey using statistical methods and evaluation of the reliability and validity of the scale draft

#### Phase 1

In Phase 1, a qualitative descriptive investigation will be conducted to create a preliminary scale.

##### Phase 1-1

A literature review will be performed to identify factors influencing eating behaviors. For this literature review, the approach by Kraus et al [27] will be followed, and PubMed and CINAHL databases will be used as literature sources. We will consider primary research articles written in English, published within the last 10 years, and available as full texts for our analysis. Keywords such as “heart failure,” “eat,” “behavior,” “diet,” “food,” “meal,” “sodium,” and “preparation” will be incorporated in the search to specifically target relevant articles in the context of this study. Additionally, references cited within the selected literature will be manually searched, and any articles identified as relevant to the eating behaviors of patients with HF will be included in the analysis based on their context or titles. The selected literature will be analyzed qualitatively and inductively. Descriptions of factors influencing the eating behaviors of patients with HF will be extracted from each source. To maintain the meaning and content, these descriptions will be standardized consistently to illustrate how various factors influence the eating behaviors of patients with HF. The extracted descriptions will be categorized based on their similarities and differences. Multiple researchers will review and verify the categorization to ensure reliability and validity.

##### Phase 1-2

Semistructured interviews with patients with HF will be conducted to gather insights into the factors influencing their eating behaviors. Interviews will be conducted in person according to an interview guide. Verbatim transcripts generated from the interviews will be returned to the participants for verification and to obtain their comments. Demographic and clinical information, such as age, sex, New York Heart Association (NYHA) functional classification [1], height, weight, HF history, hospitalization due to HF, other medical conditions or history, details of medications, and whether patients received nutritional guidance, will be extracted from their medical records. Information on participants’ living arrangements, employment status, perceived financial status, use of community resources, means of transportation to grocery stores, frequency of cooking meals and meal delivery services, presence of cohabitants, and essential characteristics of cohabitants will be collected using a self-administered questionnaire to obtain more data. The age distribution of the targeted patients will encompass the entire age range susceptible to HF, including a minimum of 1 participant aged <50 years, 1 participant in their 60s, and 2 participants each in their 70s and 80s, totaling at least 6 individuals.

Data obtained from medical records and self-administered questionnaires will be analyzed using simple aggregation. Interview transcripts will be collected verbatim and analyzed using Sandelowski’s qualitative descriptive approach [28]. The analysis will commence once the data from the 6 patients with HF have been collected. Transcript data collected verbatim from subsequent participants will be included in the analysis, and the process will continue until theoretical saturation is achieved. The analyses will be conducted independently by 2 researchers.

##### Phase 1-3

Semistructured interviews will be conducted with experts to gather insights into the factors influencing the eating behaviors of patients with HF. The interviews will be conducted following an interview guide, either in person or online. Transcripts generated verbatim from the interviews will be returned to the experts for verification and to obtain their comments. The target experts for the interviews will consist of 6 members, 1 from each specialization area:

1. Certified physicians of the Japanese Society for Clinical Nutrition, specializing in cardiology
2. Certified nurse in chronic HF care
3. Clinical nurse specialist in chronic disease care
4. Certified HF educator
5. Nutrition support team (NST)-specialized therapist
6. Visiting nurse

The NST-specialized therapist and visiting nurse will target individuals with extensive experience caring for patients with HF. Verbatim transcripts from the interviews will be analyzed using Sandelowski’s qualitative descriptive approach [28]. The analysis will be conducted independently by 2 researchers.

Based on the results obtained in Phase 1, a set of scale items and a Likert rating scale will be constructed as preliminary scales.

#### Phase 2

In Phase 2, cognitive interviews with patients with HF and experts will be conducted, using a preliminary scale to evaluate its content validity. The COSMIN guidelines recommended having a sample size of at least 7 participants for qualitative evaluation of content validity [25]. If any issues with the scale are identified through the assessments conducted in Phases 2 and 3, they will be modified as appropriate.

##### Phase 2-1

The surface validity of the preliminary scale will be assessed through semistructured interviews with patients with HF. Patients with HF will be recruited from a population different from the one used in the previous investigation. The participants will consist of at least 7 individuals, including 1 individual in their 50s and 60s and 2 individuals each in their 70s and 80s. Cognitive interviews and analysis will follow the approach proposed by Willis [29]. Cognitive interviewing allows us to understand respondents’ thought processes and identify issues with the scale. This approach involves asking participants to verbalize their thought processes while answering, allowing researchers to detect misunderstandings, ambiguities, and cognitive difficulties. This method ensures the face validity of the scale. The interview guide will be developed based on the questionnaire items created for the preliminary scale. The interviews will be conducted in person following this procedure:

1. Explaining the purpose of measuring the scale to the patients.
2. Showing patients the items on a preliminary scale and requiring them to provide written responses to each question.
3. Once responses are obtained, we will orally confirm the thought process that the patients underwent when answering the questions (think-aloud).
4. We will verbally probe and confirm the content of the question items in more detail, checking if the initial responses align (verbal probing).
5. Steps (2)-(4) will be repeated for all question items.
6. Verifying that no important content relevant to the patient is missing across the entire scale.

The verbatim transcripts obtained from the interviews will be analyzed from the following perspectives:

1. Were there any doubts or concerns regarding the content of the questions?
2. Were the intended responses obtained from the question items?
3. Were there any ambiguities or unclear language expressions in the question items or response choices?
4. Were there any deficiencies in the questions regarding important content relevant to the participants themselves?

The analysis will be conducted independently by 2 researchers.

##### Phase 2-2

The clarity, relevance, and comprehensiveness of the preliminary scale will be assessed through cognitive interviews, involving at least 7 patients with HF included in the previous investigations. The interview guide will be developed based on the questionnaire items created for the preliminary scale. The interviews will be conducted in person following this procedure:

1. Participants will read and respond to the paper-based scale.
2. Orally confirming how participants understood the content of the scale questions.
3. Verbally confirming if the scale questions were relevant to their own experiences.
4. Steps (1)-(3) will be repeated for all the question items.
5. Verifying that no important missing content in the scale is relevant to the participant’s experiences.

Transcripts will be created verbatim from the interviews, and the assessment will revolve around examining the clarity of each question item, its relevance to patient situations, and the presence of any pertinent information that might be missing. The analysis will be conducted independently by 2 researchers.

#### Phase 2-3

The relevance and comprehensiveness of the preliminary scale will be assessed through cognitive interviews with experts. The investigation will involve 7 experts, including 6 experts identified in Phase 1-3, and an additional expert who is a nursing faculty member experienced in scale development. These 7 experts will be the target participants and will be distinct from those involved in previous investigations. Interviews will be conducted either in person or online.

1. Participants will read the scale on paper.
2. The questionnaire items will be checked for their relevance to patients with HF.
3. If any questionnaire item is perceived as lacking relevance, its reasons will be investigated.
4. The overall scale will be reviewed to ensure that important content related to patients with HF is not missing.
5. The reasons for perceiving the content as insufficient will be confirmed if it was perceived as such.

Transcripts will be created verbatim from the interviews, and the assessment will revolve around examining its relevance to the patients’ situations and the presence of any pertinent information that might be missing. The analysis will be conducted independently by 2 researchers.

#### Phase 3

We will use the scale scrutinized in Phase 2 to conduct a cross-sectional study involving patients with HF. Data will be collected from the clinical records and self-administered questionnaires or scales. The items are listed in Textbox 2. A cross-sectional survey consisting of preliminary and primary investigations will be conducted. The collection of clinical records data will be conducted with the approval of the medical institution’s ethics review board. After obtaining this approval, consent from the eligible patients for their participation in the study will be requested. Clinical records will then be collected from the medical institutions where the co-researchers are affiliated or from the collaborating medical institutions, with the cooperation of both the co-researchers and collaborating researchers.

**Inventory of data obtained from the cross-sectional survey in Phase 3.**

**Clinical record**

- Age, sex, New York Heart Association (NYHA) classification [1], height, weight, heart failure (HF) history, hospitalization due to HF, other existing medical conditions, medical history, medications, and whether nutritional guidance was provided

**Self-administered questionnaire of lifestyle situations**

- Living arrangements, employment status, perceived financial status, use of community resources, means of transportation to grocery stores, frequency of cooking meals on own, frequency of using meal delivery services, presence of cohabitants, essential characteristics of cohabitants, and frequency of meals prepared by a cohabitant or home helper

**Self-administered scales**

- The preliminary Self-Administered Eating Behaviors Scale for Heart Failure (SEBS-HF)
- The Japanese version of the Medical Outcomes Study 36-Item Short-Form Health Survey (SF-36)
- The Japanese version of the European Heart Failure Self-care Behavior Scale (EHFScBS)
- Established scales for assessing criterion-related validity

### Preliminary Investigation

Data will be collected using clinical records, self-administered questionnaires, and the preliminary SEBS-HF. To confirm normality, the sample size will be approximately 30 [30]. In addition, data will be collected from patients attending collaborating medical institutions. The principal investigators collaborating at these institutions will select patients based on established eligibility criteria. Once informed consent has been obtained, the patient’s clinical records will also be collected. A survey set, which includes the self-administered questionnaire and preliminary SEBS-HF, will be provided along with a return envelope for data collection. Calculations will be made from the acquired data to determine the mean scores, standard deviations, and individual item score distributions. Normality tests will be also performed. Moreover, potential ceiling and floor effects will be examined based on the mean and standard deviation.

### Primary Investigation

The reliability and validity of the data acquired from the primary investigation will be examined using statistical methods (Table 1).

**Table 1:** Statistical analysis procedures and considerations for assessing reliability and validity.

Statistical analysis	Procedure
Descriptive statistics	Measure means, SDs, and frequencies
Normality assessment	Conduct a Shapiro-Wilk test, which calculates a test statistic based on the observed data and compares it against the Shapiro-Wilk distribution.
Item analysis	Evaluate ceiling and floor effects and perform item-total correlation analysis. Items with a correlation coefficient *r* <0.3 or >0.7 may be considered for removal or integration [31].
Internal consistency	Calculate Cronbach α coefficient. A coefficient of ≥0.7 is considered to indicate internal consistency [32].
Structural validity	Perform an exploratory factor analysis to ascertain the degree of shared variance among the items and elucidate potential factor groupings. The factor structure established through exploratory factor analysis is assessed with confirmatory factor analysis to check its fit with data from a different population.
Criterion validity	Calculate descriptive statistics for the external criterion scale and confirm normality. Then, calculate the correlation coefficient between the criterion scale and the scale under development. The validity is evaluated based on the value of the correlation coefficient.
Construct validity	Calculate the correlation coefficient between subgroups to verify hypotheses. In this scale, it is assumed that aging and the decline in physical and mental functions due to HF^a^ symptoms will affect eating behavior negatively. Therefore, verification will be done using age, NYHA^b^ classification [1], and certification of care-need level subgroups.
Measurement error	Calculate the intraclass correlation coefficient, SE, and detectable minimum change for the total scale and subscale scores obtained from the first and second tests. The sample size should be >50 participants to verify measurement error. An intraclass correlation coefficient of ≥0.7 is considered to indicate stability [33].

^a^HF: heart failure.

^b^NYHA: New York Heart Association.

In addition to the data collection methods used in the preliminary investigation, the primary investigation will incorporate the test-retest method to assess measurement error and establish scales to evaluate criterion-related validity. In the test-retest method, participants will initially respond to the SEBS-HF and then provide their responses again 2 weeks later. To assess the criterion-related validity of the SEBS-HF, we will select the Medical Outcomes Study 36-Item Short-Form Health Survey [34] and the EHFScBS [8] as external measures. We will request the study participants to provide their responses to these instruments. If more suitable external measures are available when the survey commences, we may opt to use them.

The maximum likelihood method and promax rotation will be used in the exploratory factor analysis. The factor loadings obtained will enable commonality assessment among the questionnaire items. Items with a factor loading exceeding a certain threshold will be selected. Consideration will be given to the excluded items that did not meet this threshold or loaded on multiple factors. If items are excluded, a factor analysis will be performed again.

The factor structure established by exploratory factor analysis will be examined to fit the data collected from different populations. This will be performed using goodness of fit indices (goodness-of-fit index, adjusted goodness-of-fit index, comparative fit index, and root mean square error of approximation) to evaluate the appropriateness of the model. If the fit does not meet specific standards, a review of the questionnaire items or reconsideration of the exploratory factor analysis will be needed.

According to the COSMIN guidelines [24], the sample size should be 5-7 times the number of scale items and not <100. Assuming the scale had 30 items, we planned to include more than 150 participants in the primary investigation. Data from different populations are required to conduct exploratory and confirmatory factor analyses. Therefore, the total sample size should exceed 300 participants.

### Ethical Considerations

In Phases 1 and 2, before starting interviews of patients with HF and experts, the research participation details will be explained using a document and written informed consent will be obtained. Consent can be withdrawn within certain time limits by signing and returning a withdrawal form or by email. As compensation for participating in the research, patients with HF received a gift card worth approximately US $20, and experts received a gift card worth approximately US $35 upon the completion of each interview.

In Phase 3, when distributing the set of survey forms to the eligible patients with HF, the research participation details will be explained using a document, and consent will be obtained in a similar manner. The set of survey forms will include a small gift as a token of appreciation for participating in the research.

The Ethics Review Board of the Graduate School of Nursing at Sapporo City University approved this study. The first phase of the investigation had already been approved by the Ethics Review Board (No. 4, 2023) on August 30, 2023. This study will be conducted in accordance with the principles outlined in the Declaration of Helsinki.

## Results

In the first phase of this study, a literature review was conducted to extract factors influencing eating behavior in patients with HF. On January 26, 2024, a comprehensive search using keywords such as “HF,” “diet,” “eating behavior,” “meal prep*,” and “culinary” was performed in databases including PubMed and CINAHL. This search yielded a total of 4145 articles. Relevant literature was selected based on the examination of titles and abstracts to extract the factors influencing eating behavior among patients with HF.

As of May 2, 2024, a total of 7 patients with HF and 6 experts have already been enrolled as study participants.

The draft creation of the scale is scheduled to be completed in 2024, and the evaluation of the content validity of the draft scale is expected to be finished by early 2025. The third phase will begin its investigation in mid-2025 and is expected to be completed by late 2025, after which the SEBS-HF will be published.

## Discussion

### Expected Findings

This study aims to develop the SEBS-HF to evaluate the eating behaviors of patients with HF living at home. This section discusses how SEBS-HF development might improve the eating behaviors of patients with HF living at home.

Given the concerns surrounding the relationship between malnutrition and adverse outcomes in patients with HF, proper dietary management has emerged as a pivotal factor in enhancing prognosis [35].

Minimal barriers to eating are desirable to maintain appropriate dietary management. According to the ICF framework, negative aspects of body functions and structures, environmental, and personal factors, can affect eating behaviors, which are included in the activities and participation domains. In other words, characteristics specific to heart failure patients, such as frailty [36], depression, and social isolation [37], are expected to influence eating behaviors negatively. Therefore, the SEBS-HF will be designed to quantitatively evaluate and assess the impact on eating behaviors specific to heart failure patients through self-administered evaluations.

Through self-administered evaluations, patients can identify their strengths and weaknesses. Additionally, sharing the evaluation results from the SEBS-HF with medical and welfare professionals can pave the way for individualized patient-centric support. Such assistance enhances the patient’s ability to manage their diet appropriately and leads to improved well-being.

Furthermore, as the SEBS-HF is quantified using a Likert scale, if a correlation between the results and adherence to dietary habits and readmission due to acute decompensated HF becomes evident, it could serve as a potent predictor.

Notably, a significant number of patients with HF may have dementia, which affects their self-care. The SEBS-HF is specifically designed for patients with HF who manage their diet at home, without accounting for those patients who require additional assistance because of conditions such as dementia.

### Limitations

This study will verify the reliability and validity in accordance with the COSMIN guidelines [24,25], but there are some limitations. First, a longitudinal study must evaluate the responsiveness of the measure or the process of validating the ability of the instrument to detect changes in the measured construct over time. Second, interpretability, which implies setting cutoff values, will not be measured. Following SEBS-HF development, we aim to evaluate the clinical importance of factors such as adherence to dietary habits and readmission due to acute HF to determine appropriate cutoff points. Finally, the cross-cultural validity of the SEBS-HF, explicitly designed for Japanese, remains to be assessed in different linguistic and cultural contexts.

### Conclusions

The development and use of this scale will enable a more comprehensive evaluation of the factors influencing eating behaviors in patients with HF. Patients will be able to conduct self-assessments using this scale. Sharing the results with health care professionals will allow patients to identify eating behavior issues, potentially improving dietary adherence. Therefore, medical and welfare professionals will provide appropriate support tailored to their patients’ needs.

## Abbreviations

BDPS: Brief Dietary Psychosocial Scale
BIRD: Burden Scale in Restricted Diets
COREQ: Consolidated Criteria for Reporting on Qualitative Research
COSMIN: Consensus-Based Standards for the Selection of Health Measurement Instruments
DSRQ: Dietary Sodium Restriction Questionnaire
EHFScBS: European Heart Failure Self-care Behavior Scale
HF: heart failure
ICF: International Classification of Functioning, Disability, and Health
NST: nutrition support team
NYHA: New York Heart Association
SCHFI: Self-Care of Heart Failure Index
SEBS-HF: Self-Administered Eating Behaviors Scale for Heart Failure

## Data Availability

The data collected or analyzed during the course of this study will not be made available to third parties. However, upon reasonable request, associated questionnaires and protocols used in the study may be provided by the corresponding author.
